# Utility of Remimazolam for Fast-Track Recovery Following Surgical Aortic Valve Replacement in an Elderly Patient With Severe Aortic Stenosis: A Case Report

**DOI:** 10.7759/cureus.55812

**Published:** 2024-03-08

**Authors:** Yumi Obata, Yusuke Seino, Mako Takeda, Miki Sakamoto, Soichiro Inoue

**Affiliations:** 1 Anesthesiology, St. Marianna University School of Medicine, Kawasaki, JPN

**Keywords:** delirium, fast-track, cardiopulmonary bypass, aortic valve stenosis, remimazolam

## Abstract

Remimazolam is an ultra-short-acting benzodiazepine that has minimal hemodynamic effects and is useful for early extubation after cardiac surgery. We present a case of an elderly patient with severe aortic stenosis (AS) who underwent surgical aortic valve replacement (AVR), was extubated in the operating room, and recovered quickly without postoperative delirium. An 87-year-old woman with severe AS underwent AVR under cardiopulmonary bypass. General anesthesia was induced with remimazolam 10 mg over one minute and fentanyl 100 µg, and maintained with remimazolam 0.4-0.7 mg/kg/hour, fentanyl, and remifentanil. Intraoperative hemodynamic condition was stable without vasopressors. Remimazolam was discontinued after sternum closure. She recovered consciousness five minutes after the completion of the surgery, and the tracheal tube was removed in the operating room. Remimazolam may be useful for fast-track recovery following surgical AVR in an elderly patient with severe AS.

## Introduction

Early recovery and prevention of postoperative delirium and cognitive dysfunction are crucial for improving outcomes in cardiac surgery among elderly patients [1]. Intraoperative hypotension and prolonged postoperative ventilation are risk factors for postoperative delirium [2,3], for which maintenance of blood pressure and early extubation are key strategies. In particular, patients with aortic stenosis (AS) are susceptible to hypotension and hemodynamic compromise during general anesthesia. Remimazolam, an ultra-short-acting drug with minimal hemodynamic impact [4,5], may contribute to improved outcomes. We present a case of an elderly patient with severe AS who underwent surgical aortic valve replacement (AVR) under cardiopulmonary bypass (CPB), was extubated in the operating room, and recovered quickly without postoperative delirium.

## Case presentation

An 87-year-old woman (height, 140 cm; weight, 49 kg) presented with shortness of breath and heart failure due to severe AS. She was treated with a diuretic and non-invasive positive pressure ventilation for heart failure. She was on antihypertensives and oral diabetes medication for hypertension and diabetes, respectively, and had no liver impairment or dementia. Preoperative transthoracic echocardiography showed severe AS, with an aortic valve area of 0.64 cm^2^ and mean pressure gradient of 43 mmHg, and normal left ventricular function without left ventricular hypertrophy, as evidenced by a diastolic/systolic left ventricular diameter of 46/34 mm, left ventricular ejection fraction of 51%, and interventricular septum/posterior wall thickness of 10/11 mm. Her predicted mortality derived from the European System for Cardiac Operation Risk Evaluation II (EuroSCORE II) was 28.5%. Coronary angiography showed no significant coronary lesion. Elective surgical AVR was planned on the eighth day after admission, as transcatheter aortic valve implantation was not available at the facility.

On her arrival in the operating room, her level of consciousness was normal, and vital signs were within normal limits: blood pressure, 132/76 mmHg; heart rate, 82 bpm in sinus rhythm; and oxygen saturation, 96% on room air. After the insertion of an arterial pressure line, general anesthesia was induced with remimazolam 10 mg over one minute and fentanyl 100 µg under the monitoring of the bispectral index (BIS) and regional cerebral oxygen saturation, followed by tracheal intubation. A transesophageal echocardiography probe and pulmonary artery catheter were inserted. General anesthesia was maintained with remimazolam 0.4-0.7 mg/kg/hour, remifentanil 0.05-0.25 µg/kg/minute, and intermittent administration of rocuronium. During the operation, hemodynamics and BIS remained stable. Aortic cross-clamp time, CPB time, operation time, and anesthesia time were 66, 90, 153, and 240 minutes, respectively.

**Figure 1:**
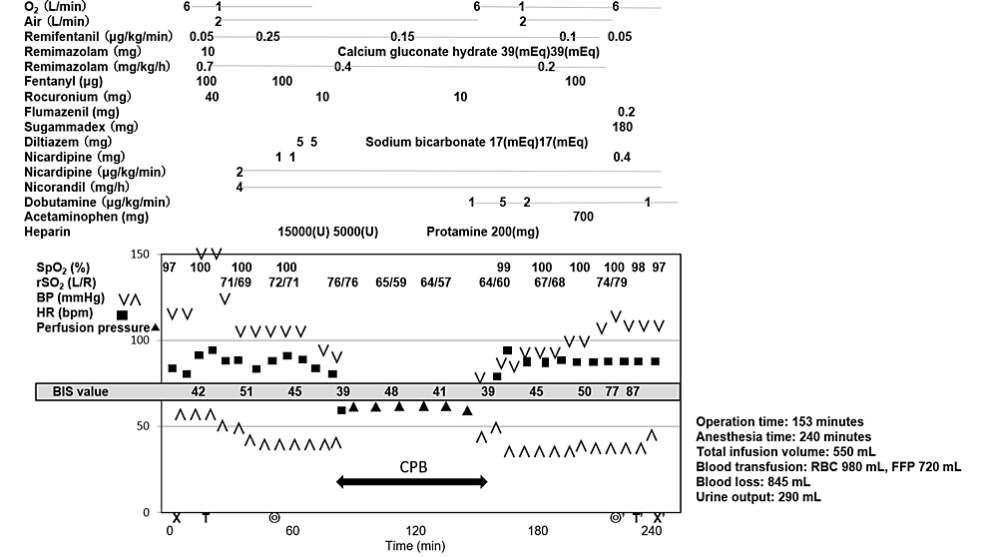
Anesthesia chart for the patient during her surgical aortic valve replacement and general anesthesia with remimazolam. At the bottom of the graph, X and X' indicate the start and end of general anesthesia, respectively; T and T' indicate tracheal intubation and extubation, respectively; and the double circle and double circle' indicate the start and end of surgery, respectively. BIS, bispectral index; BP, blood pressure; CPB, cardiopulmonary bypass; FFP, fresh frozen plasma; HR, heart rate; RBC, red blood cells; rSO_2_, regional cerebral oxygen saturation; SpO_2_, oxygen saturation.

Remimazolam was discontinued after sternum closure, and 100 µg of fentanyl and 700 mg of acetaminophen were administered. Spontaneous respiration recovered about two minutes after administration of sugammadex 180 mg, and tidal volume was ≥350 mL. Approximately two minutes later, due to persistent eye opening in response to verbal stimulation, 0.2 mg of flumazenil was prophylactically administered and she was extubated in the operating room (Figure 1).

She was transferred to the cardiac care unit (CCU) without respiratory depression, upper airway obstruction, or the need for re-sedation. After being admitted to the CCU, she woke up feeling well, understood that the operation had been successfully completed, and could communicate effectively. Six hours after admission to the CCU, she started drinking water and was able to eat on the morning of postoperative day (POD) 1. From POD 1 to POD 5, her Richmond Agitation-Sedation Scale ranged from -2 to 0, the Confusion Assessment Method for the intensive care unit was negative for delirium, and the Intensive Care Delirium Screening Checklist scored 2 or 3 points. She was discharged from the CCU on POD 1 and from the hospital on POD 39, without experiencing delirium during her CCU and hospital stay.

## Discussion

We identified three important clinical implications from this case. First, remimazolam has the potential as a suitable agent for general anesthesia in patients with severe AS due to its minimal hemodynamic effect. Second, remimazolam may facilitate the safe early extubation of elderly patients in the operating room, even following surgical AVR under CPB. Third, remimazolam might also contribute to early recovery in elderly patients without postoperative delirium.

Remimazolam, an ultra-short-acting benzodiazepine, has received approval in Japan as a sedative for general anesthesia. The recommended dosage of remimazolam is 6-12 mg/kg/hour until the loss of consciousness for the induction of general anesthesia, with a maintenance dosage ranging from 0.2 to 1.0 mg/kg/hour. However, lower doses have been reported for patients with severe AS undergoing AVR or transcatheter aortic valve replacement (TAVR) [4,5]. In a retrospective single-center analysis, remimazolam was administered at a dose of 0.18 mg/kg (interquartile range: 0.16-0.22 mg/kg) and 0.48 (0.30-0.55) mg/kg/hour for the induction and maintenance of general anesthesia in patients with severe AS undergoing TAVR. The utilization of remimazolam resulted in less overall vasopressor usage when compared to conventional general anesthetics [4].

When used in conjunction with remifentanil, remimazolam has been reported to have a lower incidence of hypotension and reduced use of vasopressors compared to propofol [6]. The incidence of hypotension was similar in the American Society of Anesthesiologists Physical Status Class III patients, indicating that remimazolam can be used safely in critically ill patients [7]. In contrast, propofol, when combined with remifentanil, can induce bradycardia and hypotension and should be administered with caution in critically ill patients [8,9]. Remimazolam may be a suitable choice for the hemodynamic management of patients with severe AS because it helps prevent excessive vasodilation, which can result in severe hypotension [4,5]. In the present patient, total intravenous anesthesia with remimazolam was chosen to avoid excessive vasodilation. As a result, blood pressure was maintained without the need for vasopressors during the induction and maintenance of general anesthesia and throughout CPB.

Remimazolam is rapidly and primarily hydrolyzed by hepatic carboxylesterase 1, without forming less active metabolites [10]. The context-sensitive half-time of remimazolam is 15-17 minutes [11,12], comparable to that of propofol. Remimazolam is primarily metabolized in the liver and may exhibit prolonged effects in patients with severely impaired hepatic function, but it does not have hepatotoxicity and can be used without issues in patients with impaired renal function [10]. Therefore, remimazolam appears to be valuable for fast-track anesthesia in elderly patients, especially those with potential organ dysfunction. The present patient was promptly awakened and extubated in the operating room without experiencing prolonged effects of remimazolam, even after undergoing cardiac surgery with the use of CPB.

Elderly patients after cardiac surgery are at a high risk for postoperative cognitive dysfunction and delirium. Mechanical ventilation has been reported as a risk factor for delirium [3], and fast-track recovery with early extubation and prompt CCU discharge is considered one of the strategies to mitigate delirium [13]. The use of a benzodiazepine such as midazolam is considered a risk factor for postoperative delirium due to its prolonged action [14]. In contrast, remimazolam may be less likely to cause delirium due to its short duration of action [11,12]. Furthermore, although the appropriate intraoperative perfusion pressure during CPB has not been clearly defined, a decrease in regional cerebral oxygen saturation to 50% or less due to low perfusion leads to postoperative cognitive dysfunction and prolonged hospital stay [15,16]. The potential of remimazolam to prevent intraoperative hypotension and reduced cerebral blood flow, along with its ability to facilitate early extubation, may contribute to a reduction in the occurrence of postoperative delirium and cognitive dysfunction.

## Conclusions

The present case implies remimazolam’s potential as a suitable agent for general anesthesia in patients with severe AS due to its minimal hemodynamic effect. The ultra-short-acting duration of remimazolam could potentially facilitate the safe early extubation of elderly patients in the operating room even after surgical AVR under CPB. In addition, while remimazolam might contribute to early recovery in elderly patients without postoperative delirium, definitive conclusions should await further clinical trials.
